# Total mandible and bilateral TMJ reconstruction combining a customized jaw implant with a free fibular flap: a case report and literature review

**DOI:** 10.1186/s40902-023-00374-w

**Published:** 2023-01-23

**Authors:** Flavio Andrea Govoni, Nicola Felici, Matteo Ornelli, Vincenzo Antonio Marcelli, Emilia Migliano, Bruno Andrea Pesucci, Roberto Pistilli

**Affiliations:** 1 Unit of Maxillofacial Surgery, San Camillo-Forlanini Hospital, Rome, Italy; 2 Unit of Plastic and Reconstructive Surgery of the Limbs, San Camillo-Forlanini Hospital, Rome, Italy; 3 Department of Plastic and Regenerative Surgery, San Gallicano Dermatological Institute IRCCS I.F.O, Rome, Italy

**Keywords:** Mandibular reconstruction, Free fibular flap, TMJ prosthesis, Bone fibrous dysplasia, Virtual surgical planning, Patient-specific surgery, Computer-aided design, Rapid prototyping

## Abstract

**Background:**

The need for whole mandibular bone reconstruction and bilateral joint replacement is fortunately rare, but it is an extremely challenging topic in maxillofacial surgery, due to its functional implications. CAD-CAM techniques development has opened new broad horizons in the surgical planning of complex maxillofacial reconstructions, in terms of accuracy, predictability, and functional cosmetic results. The review of the literature has revealed a small number of scientific reports on total mandibulectomy including the condyles, with only eleven cases from 1980. Most of the works describe reconstructions secondary to dysplastic or inflammatory diseases affecting the lower jaw. The aim of this work, reporting a rare case of massive fibrous dysplasia of the whole mandible, is to share our experience in the management of extended mandibular and bilateral joint reconstruction, using porous titanium patient-specific implants.

**Case presentation:**

The authors present a 20-year-old male patient suffering from massive bone fibrous dysplasia of the mandible. The mandibular body and both the rami and the condylar processes had been involved, causing severe functional impairment, tooth loss, and facial deformation. The young patient, after repeated ineffective conservative surgical treatments, has required a biarticular mandibular replacement. Using virtual surgical planning (VSP) software, the authors, in collaboration with medical engineers, have created a custom-made original titanium porous mandibular implant, suspended from a bilateral artificial temporomandibular joint. The mandibular titanium implant body has been specifically designed to support soft tissues and to fix, in the alveolar region, a free fibular bone graft, for delayed dental implant prosthetic rehabilitation.

**Conclusion:**

The surgical and technical details, as well as the new trends in mandibular reconstructions using porous titanium implants, are reported, and discussed, reviewing literature reports on this topic. Satisfactory functional and cosmetic restorative results have been obtained, and no major complications have occurred. The patient, currently in the 18^th^ month clinical and radiological follow-up, has recently completed the functional restoration program by an implant-supported full-arch dental prosthesis.

## Background

Mandibular reconstruction using free vascularized bone or composite flaps is a widely discussed topic in maxillofacial surgery. In 1991, Urken [1] published one of the first and more extensive reviews of the literature on oro-mandibular reconstructions, pointing out surgical details on flap choice to improve anatomic and functional restoration of the mandible. The free fibular flap (FFF), introduced by Hidalgo in 1989 [2], is used as the first choice, either bone or composite flap, for long segmental mandibular reconstruction in oncologic surgery [3]. Iliac crest and scapular free flaps are also widely used for shorter segmental reconstructions, as a second-choice flap [4], by surgeon’s personal preferences [5], or in pediatric patients [6]. The development of CAD-CAM techniques has opened new broad horizons in the surgical planning of complex mandibular reconstructions, introducing patient-specific titanium surgical implants that can be used to support the free vascularized bone flaps, improving mandibular shape and jaw function outcomes [7]. Virtual surgical planning (VSP) has become, in recent years, the best option to produce surgical cutting guides for bone resection, flap harvesting, fixation plates, TMJ prostheses, and patient-specific bone replacement implants [8]. These procedures, increasing the accuracy of the reconstruction, are useful tools in maxillo-mandibular replacement surgery [9], and in immediate or delayed dental implant rehabilitations [10]. The authors have reviewed the literature, from 1980 to date, searching for total mandibulectomy. Only eleven cases of bicondylar mandibular reconstructions have been reported and just two of them had required also bilateral TMJ prosthetic replacement. The need for a bicondylar mandibular reconstruction combined with simultaneous bilateral joint replacement is a very challenging topic, fortunately rare, mainly secondary to traumatic injuries, osteomyelitis, bone dysplasia, or neoplasms [11–22]. This work aims to share our surgical experience reporting a case of mandibular bone reconstruction combined with bilateral prosthetic TMJ replacement. To achieve such a complex reconstruction, we have used an original biarticulated patient-specific titanium implant, specifically designed to support a free fibular flap for delayed dental implant placement. The bone implant has been CAD-designed by the surgeons for simultaneous bilateral replacement of both the TMJ and the rami to create a mandibular body that could support, in the alveolar region, a free fibular bone flap. The original bone titanium implant has been then implemented by the engineers, before being manufactured by CAM-techniques. During the surgery the bone flap has been segmented using 3D-printed surgical guides and then it has been fixed by screws, on the titanium mandibular body, creating a new alveolar bone ridge, for delayed dental restoration.

## Case presentation

**Fig. 1:**
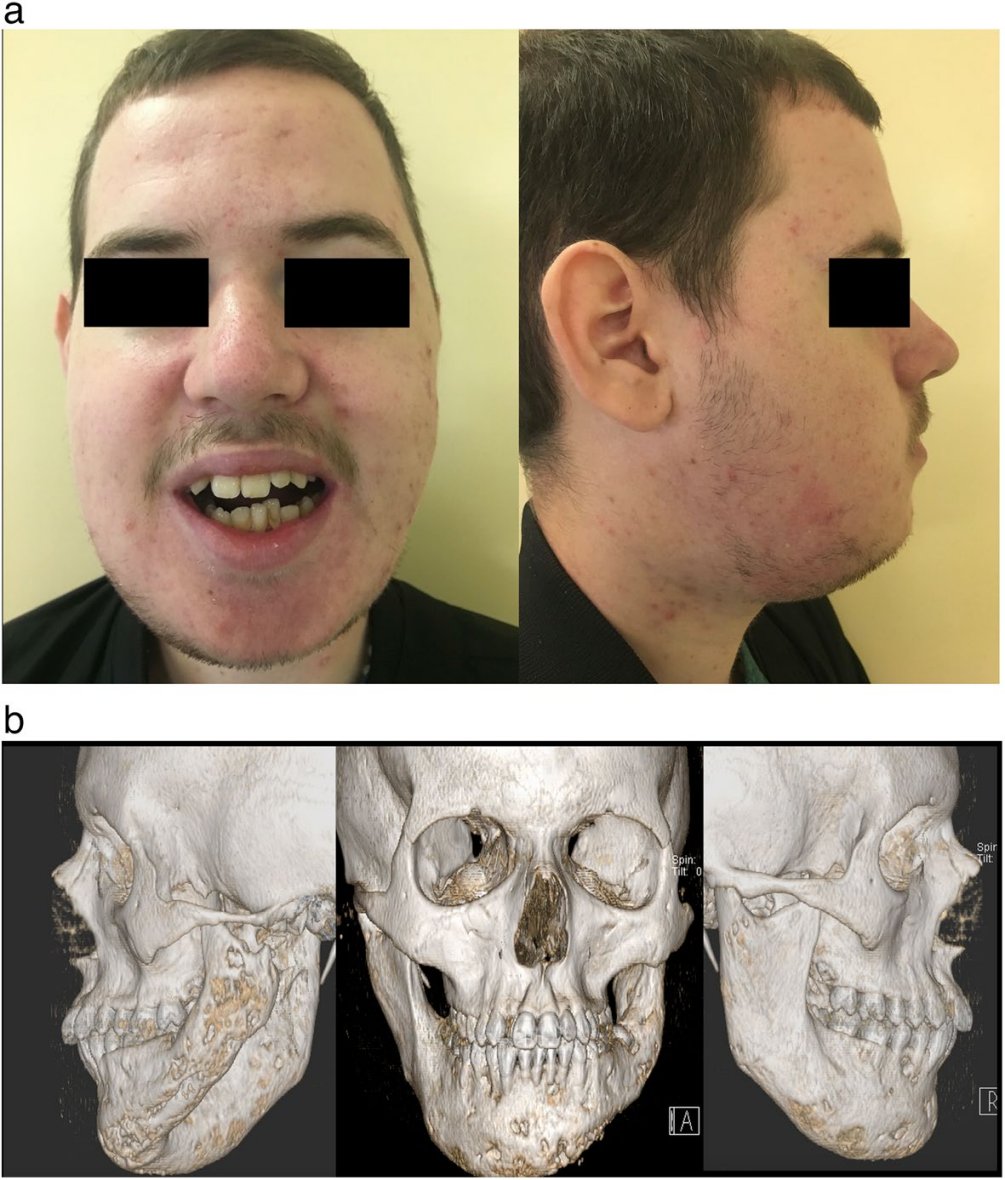
**a** 20-year-old male patient affected by massive fibrous dysplasia of the mandible, facial deformity, and severe mouth opening restriction. **b** CT3D scans in front and lateral views

A 15-year-old male patient, in 2015, was admitted to our department, reporting lower jaw progressive bulking, increasing facial deformity, chewing pain, and mandibular impairment with limited mouth opening (Fig. 1). Already operated in other hospitals, in his country of origin, he came to our observation with a radiological and histological diagnosis of fibrous dysplasia of the mandible, started when he was an 8-year-old child. Mandible multifocal biopsy confirmed the histological pattern of a massive fibrous dysplasia. The dysplasia affected the whole mandible involving both basal and alveolar bone, producing a significative deformation of the left coronoid and condylar processes, associated to a left TMJ severe dysfunction.

Our first surgical conservative approach, in 2015, consisted of mandibular bone intraoral debulking and reshaping. On the second hospital admission, 2 years later, for severe mouth opening limitation, we performed a left coronoidectomy and extraoral left condylectomy, with good temporary functional and aesthetic results. The patient, who had refused any kind of medical treatment, presented a new progressive mandibular deformation of the contralateral side developing a right condylar enlargement. Three years later, in 2020, experiencing recurrent gingival and dento-alveolar abscesses, unstable teeth, jaw pain worsening, and severe mouth opening limitation, he required a more radical surgical treatment. The literature review on bicondylar replacement of the mandible, from 1980 to date, produced only a small number of reports (eleven articles — see Tables 1 and 2). In most cases, a free fibular flap (FFF) has been used to reconstruct the mandible, shaping the bone extremities for condylar replacement or using commercial TMJ prostheses. In two cases, a whole titanium patient-specific mandibular replica has been implanted. In one case report, a freeze-dried human mandible allograft has been transplanted [11–22]. In recent years, we have been working with patient-specific porous titanium implants to give support to a FFF reconstruction in segmental mandibulectomy. In this way we achieved better cosmetic results in terms of mandibular symmetry and we managed to fix the bone flap in a more suitable position for dental implant restoration. Inspired by these results we proposed to the patient a total mandibular replacement by a free fibular flap fixed on a custom-made titanium mandibular body, bilateral TMJ prostheses, and dental implant delayed rehabilitation. Comprehensive legal informed consent to this radical surgical treatment has been acquired. High-resolution CT scans of the head and of the lower limbs with angiographic evaluation were recorded. The CT imaging dicom files were sent to an external service of medical engineering for VSP processing (see acknowledgments). The implant has been designed and manufactured to meet the following needs: (a) to replace the mandibular body and the rami with a micro-porous surface, promoting soft tissue integration; (b) to realize an internal light-weighted titanium macro-porous structure for bone tissue integration; (c) to articulate the new mandible to a bilateral TMJ cranial patient-specific prosthesis; (d) to support and fix a segmented free-fibula flap in a preplanned position, suitable for dental implant restoration.

**Table 1:** Summary table of the scientific papers reporting a “bicondylar mandibular reconstruction” — PubMed Library search results, selected and checked by a Google cross-search matching from 1980 to date (see references list from 12 to 23)

**N**	**Ref.**	**Author**	**Nation**	**Year**	**Age**	**Sex**	**Follow-up**^**a**^	**Disease**	**Bone reconstruction/joint**
1	[12]	Motlagh M. F.	Iran	2017	25	M	24	Langherans histiocytosis	Total mandible allograft/allograft condyles
2	[13]	Bhathena H.	India	1986	NR	M	12	Adenocystic carcinoma	Pectoralis major rib flap - rib neocondyles
3	[14]	Paley M. D.	UK	2005	55	F	56	Massive osteolysis	Single fibula flap - artificial Christensen joint
4	[15]	Jeremic J. V.	Serbia	2010	58	m	19	Ameloblastic carcinoma	Single fibula flap - native condyles
5	[16]	Abe T.	Japan	2011	56	M	56	Traumatic osteomyelitis	Single fibula flap - bone neocondyles
6	[17]	Winters R.	USA	2012	59	F	7	Traumatic osteomyelitis	Bilateral fibula flap - bone neocondyles
7	[18]	Poukens J.	Belgium	2012	83	F	NR	Recurrent osteomyelitis	Xilloc Ti4 prosthesis – artificial anatomic condyles
8	[19]	Grinsell D.	Australia	2014	19	M	48	Osteosarcoma	Bilateral iliac crest- bone neocondyles
9	[20]	Jambehkar S.S.	India	2015	24	F	24	Mandibular neuroma	Bilateral fibula flap - bone neocondyles
10	[21]	Rustemeyer J.	Germany	2019	50	F	12	Diffuse scler. osteomyelitis	Single fibula flap - artificial biomet joints
11	[22]	Chernohorskyi DM.	Ukraine	2021	27	F	27	Drug abuse osteomyelitis	Xilloc Ti6 prosthesis – artificial anatomic condyles

*NR* Not reported

^a^Follow-up reported in months

**Table 2:** Comparison of the different surgical solutions (see Table 1) used for bicondylar mandibular reconstructions: articular disk preservation, condylar resuspension to the glenoid fossa, soft tissue flaps or paddles for intra- or extraoral lining, functional outcomes, and dental restoration

**Author**	**Bone flap**	**Condyle**	**TMJ disk preservation**	**Mandibular body**	**Joint/material**	**Follow-up**	**Lining skin paddles**	**Mouth opening**	**Dental restoration**
Bhathena	5^th^ Rib	Bone flap	N.R.	Fractured rib unfixed	Resuspension/resorbable 2-0	24	Intraoral skin paddle	>40 mm	Dental prosthesis
Paley	1 FFF*	Prosthetic	No	Bone flap and reconstructive plate	Prosthetic Christensen	12	No	>30MM	NN.R
Jeremic	1 FFF	Native	Native TMJ	Bone flap and miniplates	Native TMJ	56	No	>30MM	N.R.
Abe	1 FFF	Bone flap	No	Reconstructive plate virtual planning	Distant Suspension	19	No	>40 mm	N.R.
Winters	2 FFF	Bone flap	Yes	Reconstructive plate virtual planning	Resuspension/not resorbalbe 0	56	Intraoral skin paddle	>30MM	N.R.
Poukens	None	Prosthetic	ND	Xilloc total mandible titanium implant	Resuspension	7	NR	>40 mm	N.R. Web - Published
Grinsell	2 DCIA	Bone Flap	Yes	Bone flap and miniplates	Resuspension/not resorbalbe 0	N.R.	Intraoral skin flap (RFFF )	>30MM	N.R.
Jambehkar	1 FFF	Bone Flap	N.R	Bone flap and miniplates	Distant suspension	48	No	>30MM	Implants/overdenture
Motlagh	Human allograft	Transplant	Yes	Allograft (cadaveric mandible)	Resuspension/resorbable 2-0	24	No	>40 mm	N.R.
Rustemeyer	1 FFF	Prosthetic	No	Bone flap and miniplates	Prosthetic biomet	12	Extraoral skin paddle	>40 mm	Implants/overdenture
Chernohorskyi	None	Prosthetic	N.R.	Xilloc total mandible titanium implant	Resuspension/NR	27	No	Good	Mobile denture

*Abbreviations*: *FFF* Free fibular flap, *DCIA* Deep inferior iliac artery, *RFFF* Radial free forearm flap, *NR* Not reported

### Implant design and additive manufacturing

**Fig. 2:**
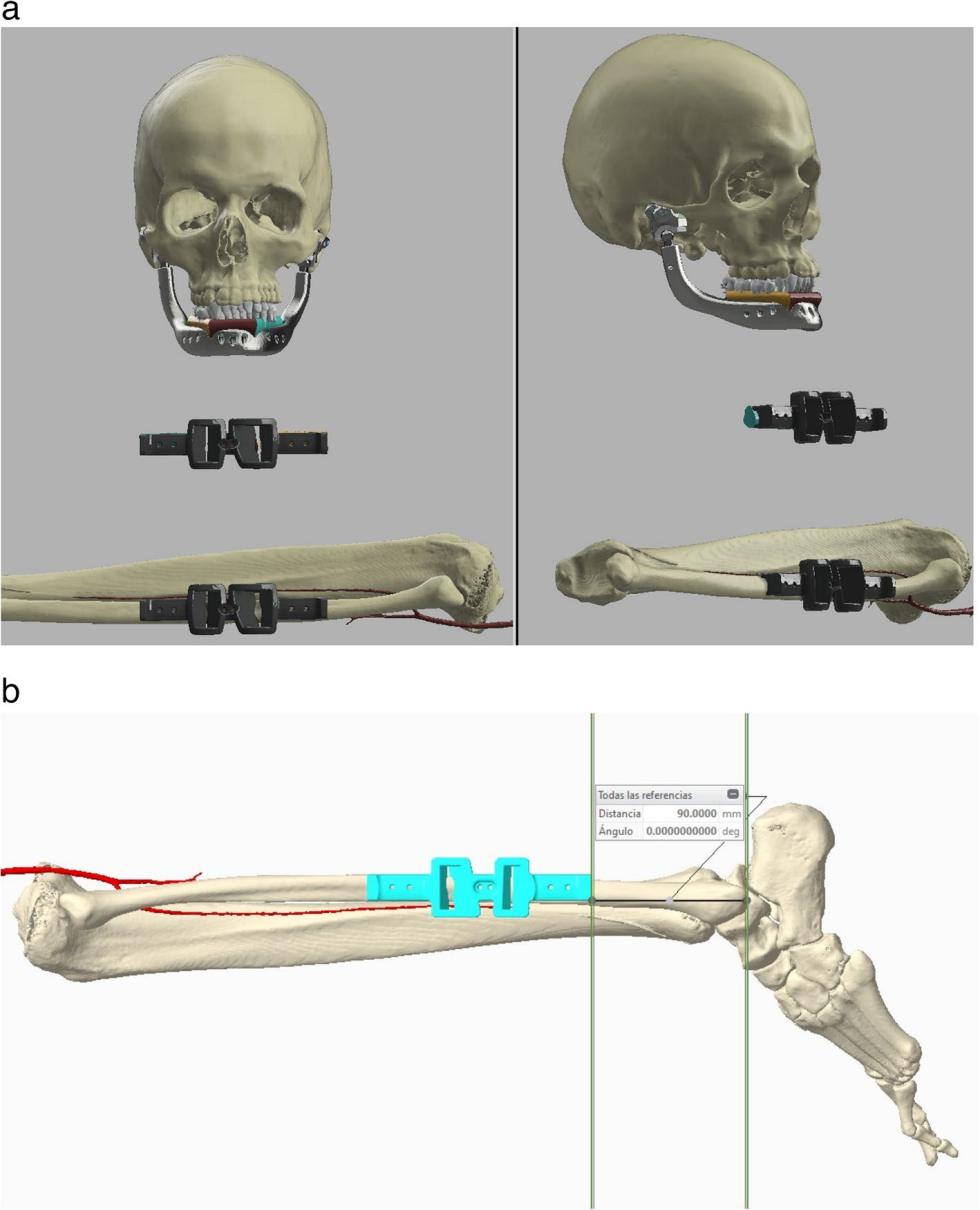
**a**, **b** Virtual surgical planning (VSP) of a free fibular flap (FFF) surgical harvesting with cutting guides prototyping, design of a mandibular body biarticulated porous titanium implant with an upper groove for bone graft insetting

**Fig. 3:**
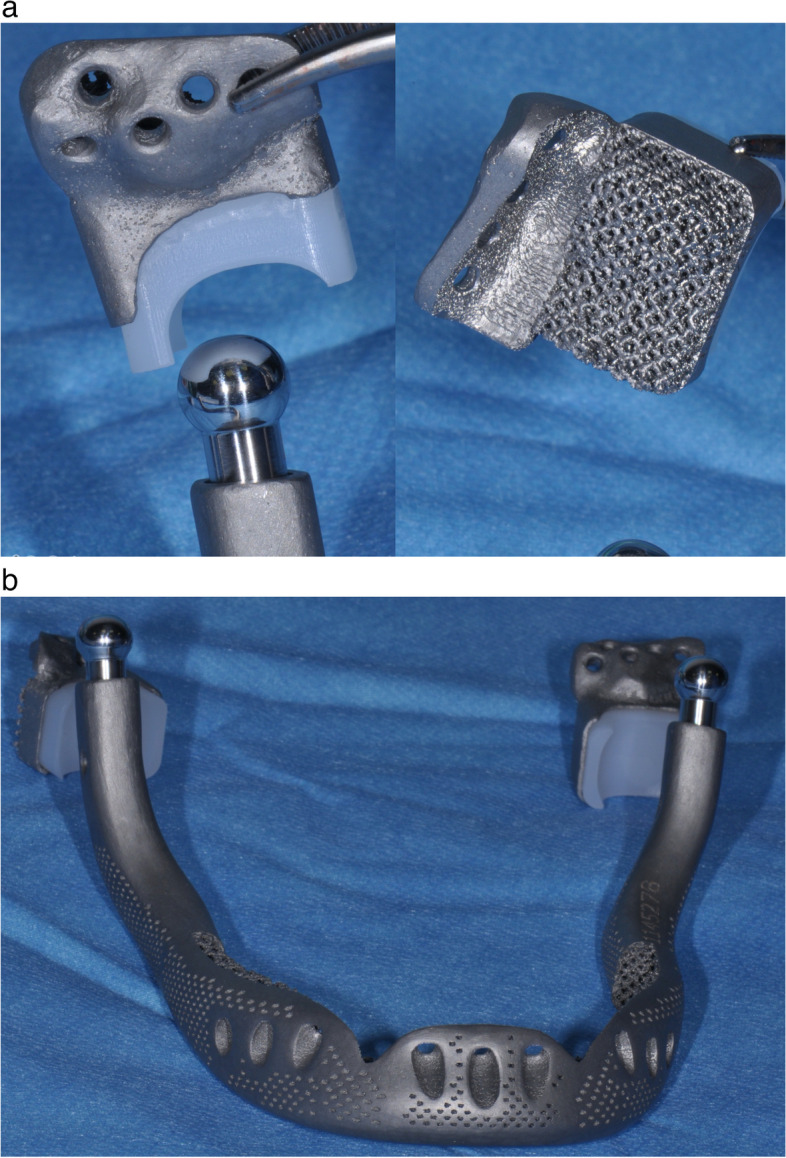
**a** TMJ prostheses: condylar and cranial components, note the porous cranial surface. **b** The whole implant provided of condylar heads on the surgical field

Digital computer-aided design (CAD) and virtual surgical planning (VSP) processes have been used to preview a total surgical biarticular mandibular resection and to plan a 3D simulation for the free fibular flap harvesting. VSP, implant design, and manufacturing have been developed in collaboration with an external engineering service, specialized in patient-specific bone implants (see acknowledgements for details). A high-resolution computer tomography of the head and of the lower limbs of the patient was reconstructed using an open-source software named “In Vesalius” (developed by Centro de Tecnologia da Informação Renato Archer, Brazil). In consecutive web-meetings, the surgeons and the engineers have discussed together about surgical needs, techniques, and patient-specific implant solutions, sharing the projects on a smartphone application “Autodesk Fusion 360™” for offline work and implementation. At the end of the project phase, the implants and the cutting guides, designed using a CAD parametric software (CREO® by PTC), were exported in “STL” format for the prototyping printing process (Fig. 2). The customized titanium mandibular body, the TMJ implants together with the bone cutting guides, have been realized by additive manufacturing. The manufacturing process used for metallic parts (implants and guides) was the Electron Beam Melting technique (EBM S12) developed by Arcam AB (Krokslätts Fabriker 27A, SE43,137 Mölndal, Sweden). This 3D metal printing device built the implants layer-by-layer (every 70 μm) using standard Ti6Al4V-ELI powder (provided by ARCAM) with an average particle diameter of 50 μm. The process took place in a vacuum (pressure of 1×10^−5^ mbar throughout the entire build cycle) and at a high temperature (800 °C). The parts produced with the EBM process did not need any thermic treatment, were free from residual stresses, and had a microstructure free from martensitic structures. The process adopted for the elimination of not melted metal volume was sandblasting with the same Ti6Al4V-ELI powder (granulometry around 50–60 µm). Supports removal and surface polishing were done manually. The final surface has been sandblasted by corundum particles. Temporo-mandibular articular prostheses have been made modular to allow future surgical revisions. The condylar heads were projected in a spherical shape and made of Co-Cr (chrome-cobalt) alloy while the cranial component has been made of UHMWP (ultra high molecular weight polyethylene), both components, machined by numeric control machines (Fig. 3). Cutting guides for fibula were crafted by polyamide powder in a SLS (selective laser sintering) process (EOS P396). Finally, all the crafted materials, instruments, and implants were cleaned in a 40-minute-long double hot ultrasonic rinsing bath (80 °C) with pure distilled water after neutral pH soap cleansing. Patient-specific 3D-printed implants, in EU countries, are subject to the Medical Devices Regulation directive EU 2017/745. A manufacturer declaration is mandatory for clinical use specifying that the product and its productive process respect European medical device fabrication rules, but patient-specifc implants do not need national authority approval or clinical trials before surgical implantation.

### Surgical treatment

**Fig. 4:**
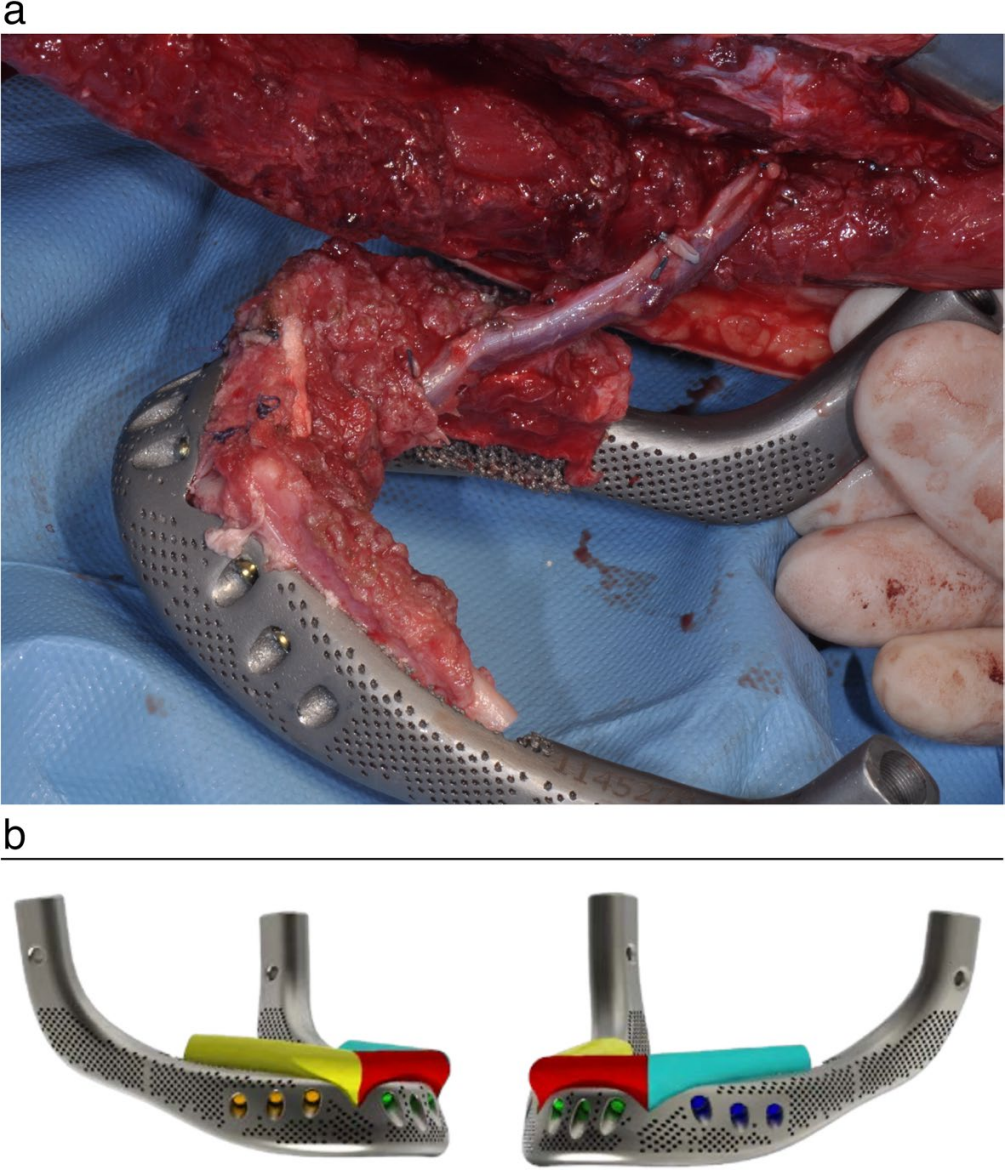
**a** The hybrid (bone/metallic) mandibular implant assembled directly at the donor site surgical field. **b** The VSP implant simulation. The free bone graft segmented in different colors and screwed in position

**Fig. 5:**
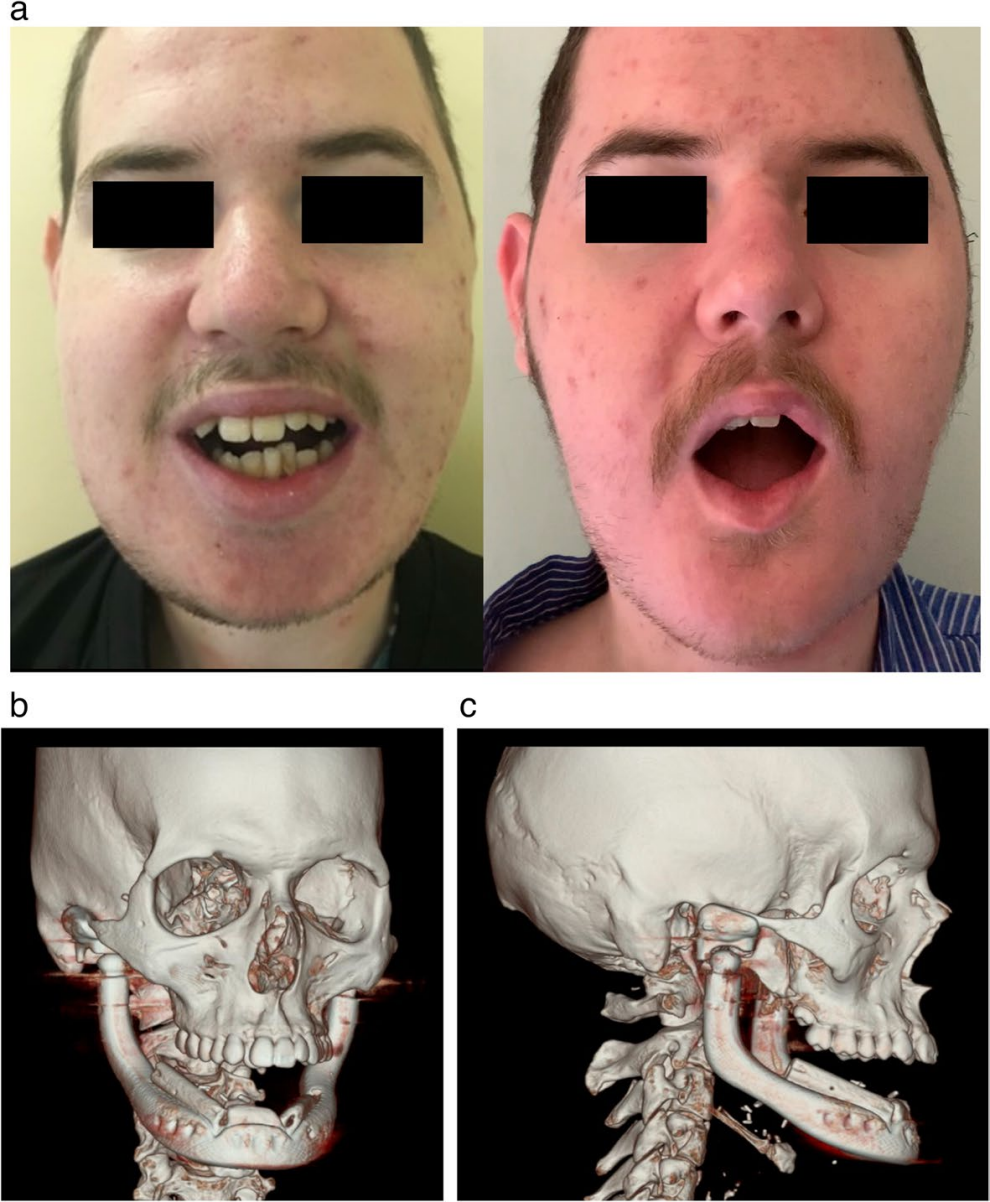
**a** Early outcomes, at 12-months follow-up. In frontal view, note the mouth opening and the facial symmetry restoration. **b**,** c** the post-operative 3DCT scan evaluation

**Fig. 6:**
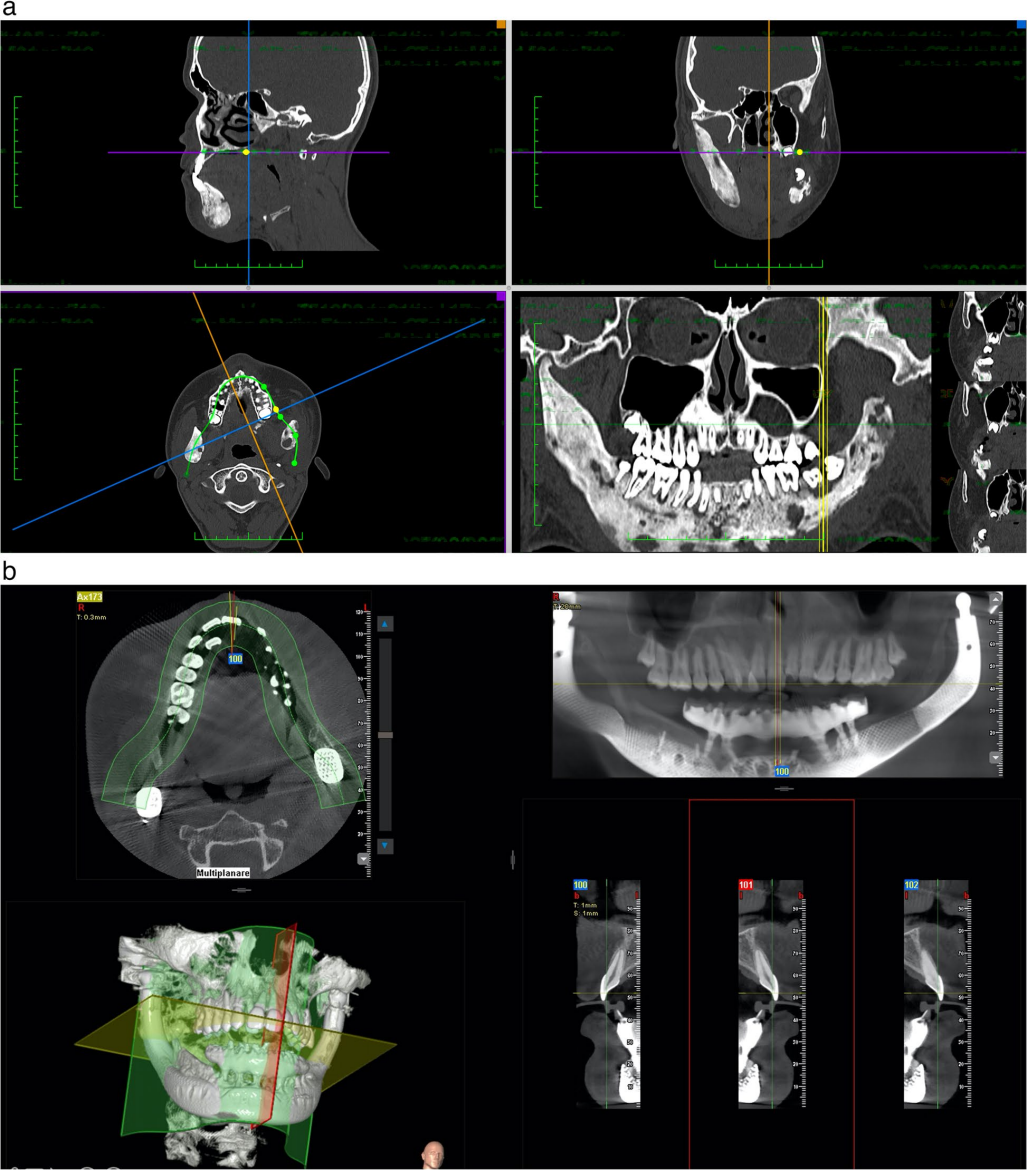
**a** Pre-operative CT scans. **b** Post-operative CT scans at the 18^th^-month follow-up after dental implant rehabilitation

**Fig. 7:**
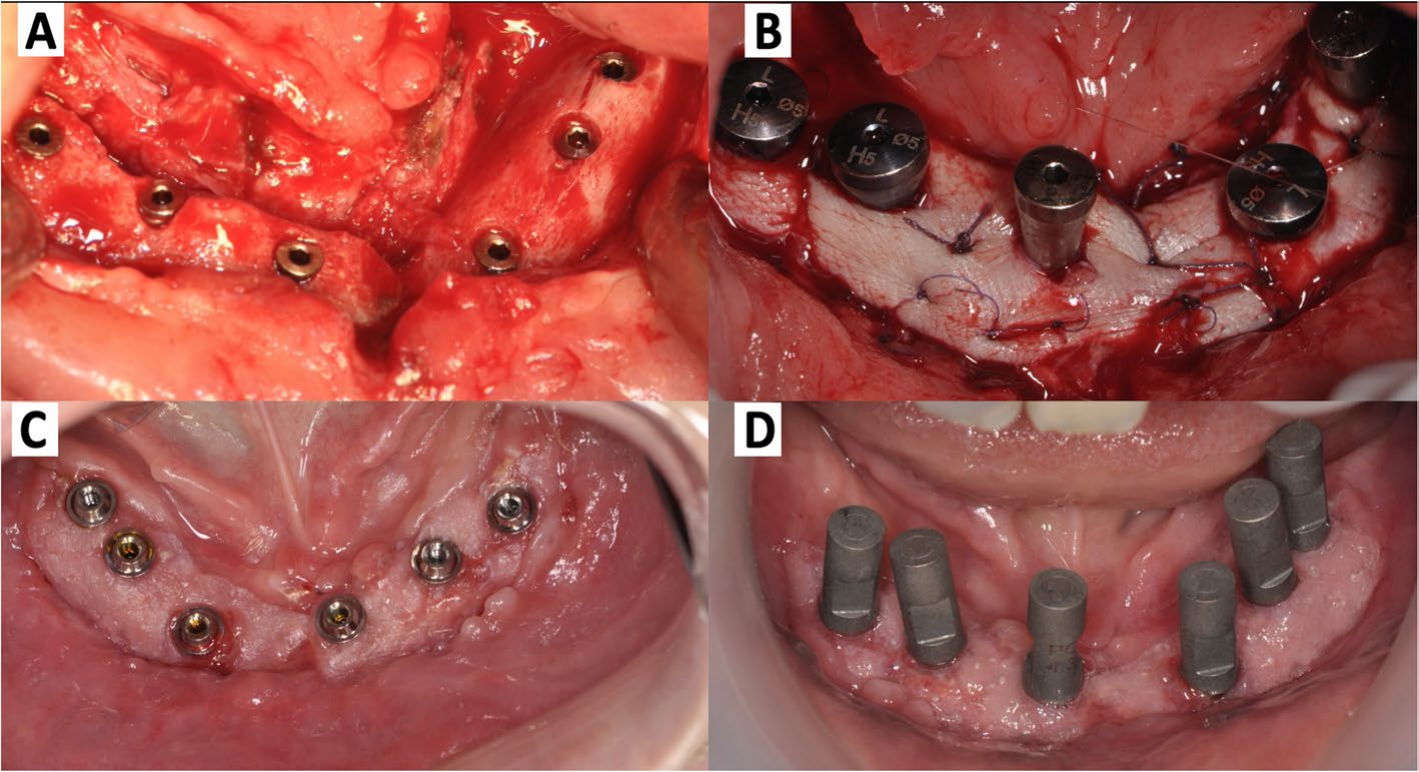
Intraoral views during the dental implant rehabilitation **a** implant placement, **b** healing screws placement and gingival reconstruction by skin grafting, **c** the results after the placement of transmucosal abutments, and **d** intraoral digital prosthetic impression of the scan bodies

**Fig. 8:**
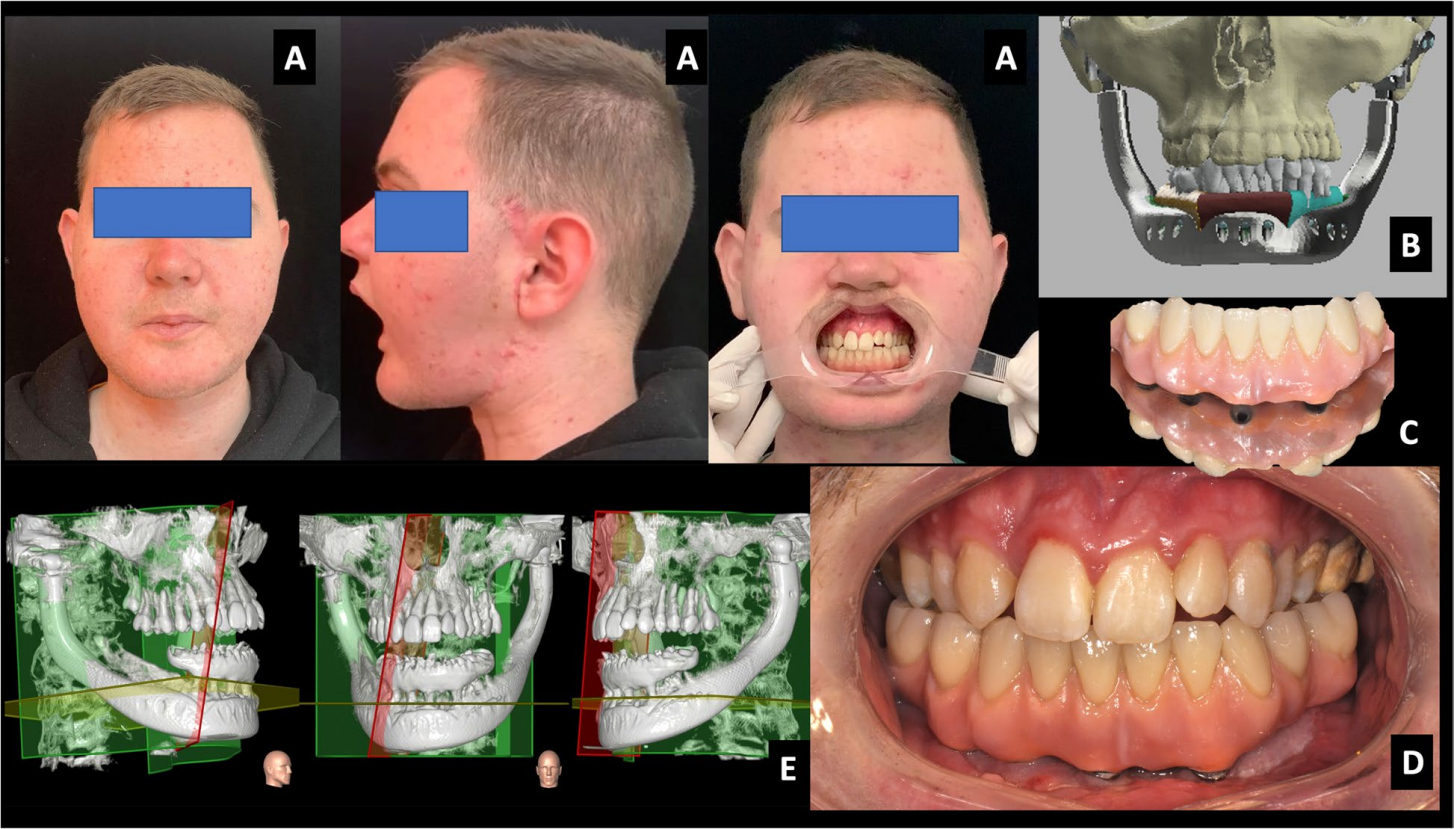
The final result at the 18^th^-month follow-up **a** patient views, note the mouth opening in lateral view and the dental occlusion, **b** the pre-treatment 3D-VSP (virtual surgical plan) in comparison with the obtained results, **c** the final dental prosthesis, **d** the 3D CT scan at the end of the treatment, and **e** the final occlusal view

The surgical operation has been realized in a one-step procedure of combined extra-intraoral mandibular and bilateral joint resection with immediate reconstruction using a hybrid FFF/titanium body biarticulated mandible (Fig. 4). Under general anesthesia, via nose-tracheal intubation, the maxillofacial team accessed and skeletonized the mandible through a Visor Flap, combined with an intraoral gingival sparing incision. The temporo-mandibular joints were approached by a preauricular incision with deep temporal plane release. TMJ dissection, exposing the zygomatic arch and the joint capsule, proceeded to the disk for condylar detachment. On the left side, where condylectomy had been already realized 5 years before, the surgical approach was directed to expose the glenoid fossa and to create a deep undermining to reach the mandibular ramus. A transcervical total resection and disarticulation of the mandible was finally obtained, after a transoral bilateral temporalis muscle release, on a subperiosteal plane. Mental and inferior alveolar nerves were cut bilaterally. The plastic surgery team approached, at the same time, the left fibula, isolating the bone flap using a traditional dissection technique, and harvesting it on the peroneal vessels, as planned in the VSP. Cutting guides have been used to obtain, directly on the donor site, three segments of the bone and to predrill the holes for bone screw fixation on the titanium mandibular implant. The FFF was then adapted and fixed by Lorenz 2.4 screws on the titanium hybrid mandible while its vascular pedicle was already connected to the patient leg vessels (Fig. 4a). Implant site preparation consisted of suitable cervical vessels isolation on the right side, right submandibular gland excision, selective ostectomies of the left temporo-mandibular fossa, fixation to the temporal bones of the two TMJ patient-specific cranial implants. The maximum length of the screws has been pre-determined by the VSP on the base of the temporal bone width. The condylar prosthetic heads have been projected as modular parts of the implant, fixed on the implant body through a “Morse cone” conical connection. Three different neck heights (from 2 to 6 mm) for condylar heads were provided to choose the intraoperative best fitting head to the glenoid fossa component. The composite implant, with its pedicle, was then positioned and revascularized on right neck vessels by end-to-end microvascular anastomoses. On the right TMJ side a 4-mm condylar head neck was used, while on the left side, the 6 mm condylar head neck demonstrated a better fit to avoid a moderate left TMJ tendency to anterior luxation. Bilateral silicone suction drains have been placed proximally to the joints and in the submandibular spaces. Broad-spectrum antibiotics were administered post-operatively, together with enteral feeding. The patient was managed for 48 h in a post-operative intensive care unit. Early outcomes, at 1, 6, 12, and 18 months after surgery, have been recorded demonstrating good functional mandibular recovery for speech and chewing, good aesthetic results in terms of facial symmetry, facial mimics, resolution of pain symptoms, and bone preservation. No major complications have been registered (Figs. 5, 6, and 7). The patient is to date entering the eighteen-month follow-up, at the end of the dental implant restoration procedure, with very satisfactory results (Fig. 8).

### Methods for literature review

A “PubMed-based” search, from 1980 to date, was launched using the keywords: “total” + “mandibular” + “reconstruction” +/− “TMJ prosthesis,” the authors took into consideration all the related articles. The “PubMed advanced search” found 908 works, only the papers reporting a bicondylar mandibulectomy with or without condylar head reconstruction, have been selected, reducing the number to 10 works. Finally, the same cross-search was done on “Google Search Engine,” to check if there were relevant news matching to the topic on web-media resources. The results of this cross-matching systematic analysis demonstrated that only ten scientific papers have matched the search criteria, reporting a bicondylar mandibular surgical replacement. One more case report, to our knowledge, is published only on the “Xilloc” patient-specific implant website resource, and does not have a related medical journal report sent by the author. An engineer report of the production of this implant written by L. Nichels in 2002 can be found on the journal Metal Powder Report [23]. All the scientific papers have been considered and compared [12–22]. The most important results have been summarized in Tables 1 and 2 then discussed in the next section.

## Discussion

Bone fibrous dysplasia (BFD) in most cases demonstrates to be a self-limiting disease during patient aging but, progressive growth into the third and fourth decades of life, has been reported. Malignant degeneration has been reported in 0.4% of cases [14]. Jawbones BFD may cause either facial deformity or severe malocclusion, alveolar bone deformation, tooth loss or migration, bone pain, and severe TMJ functional impairment [24]. Treatment of BFD in adults remains challenging, and long-term success is inherently unpredictable. Medical treatments using bisphosphonates, or other bone antiresorptive drugs, are proven to reduce pain, but seem to be ineffective on dysplastic bone growth. Immune target therapies are still off-label, experimental but promising treatments [25]. If conservative or minor surgical procedures are not successful, a major surgical approach with extended bone resection/reconstruction becomes mandatory. The case reported in this paper demonstrates, in our opinion, that satisfactory rehabilitation can be achieved even after a bicondylar mandibulectomy using a free bone flap combined to patient-specific mandibular and articular titanium implants. Reviewing the literature, from 1980 to date, we have found only eleven reported cases of bicondylar mandibular replacement (Tables 1 and 2) [12–23]. One of them, reported by Jeremic in 2010, was a bicondylar-sparing mandibular resection, so the bone flaps were attached directly to the native joints by plates and screws [15]. In two cases, prosthetic joints have been used to connect the bone flaps to the skull, reporting good functional results, one by Paley in 2005 with a Christensen joint [14], the other by Rustemeyer in 2019 with a Biomet joint [21]. In the remaining seven cases, the new mandible has been resuspended. In three cases only, the disk has been preserved to allow close resuspension of the bone flap extremity to the glenoid fossa. The most used bone flap was the FFF (six cases), while just in one case a bilateral iliac crest flap was performed. Motlagh described, in 2017, the use of a freeze-dried human allogenic mandible as a transplantation [12], while Poukens, in 2012, has replaced the whole mandible by a total prosthetic implant and no bone has been used [18]. The report by Pouken, available on the official website of Xilloc ™, a biomedical implant manufacturer, has been described as the “very first world’s 3D-printed total jaw reconstruction,” but to our knowledge, has never been scientifically published on a peer-reviewed medical journal. Recently Chernohorskyi [22], has published a clinical report on the reconstruction of a young female patient mandible using the titanium custom-made implant described by Poukens. All the Authors, anyway, reported good functional and stable outcomes, without major complications, considering a medium follow-up of 28 months, ranging from a minimum of 7 months to a maximum of 5 years. In all the cases of this review, the alveolar branch of the mandibular nerve has been resected bilaterally, and none have reported nerve reconstruction. In most cases, the disease affecting the mandible was osteomyelitis or low-grade dysplastic degeneration. Making a comparison to the reports of this literature review, the case we are presenting reveals some original aspects that, in our opinion, must be outlined and discussed. Our reconstruction project is based on a new original concept of creating a hybrid (bone/metallic) mandible. The basal mandibular bone and the rami have been replaced by a microporous surface and a macro-porous body titanium implant, designed to be integrated by connective soft and hard tissues, and to reattach muscular insertions, reducing at the same time the plate exposure risk. The mandibular body implant has been designed and shaped to support a screwed-free fibula flap to recreate the alveolar bone process of the mandible, as recently suggested by some surgeons [26–28]. The VSP procedure planned the ideal bone position for a delayed dental implant insertion. CAD-CAM techniques have revealed to be very effective, predictable and surgically timesaving in the treatment of this complex case. Even if we did not insert dental implants during the primary reconstruction, the bone position has allowed a satisfactory dental restoration. Porous titanium bone implants or porous patient-specific plates appear to produce better results instead of commercial plates, in term of healing times, tissue integration, and plate exposure rate [29, 30]. The implanted mandibular body has been secured to its prosthetic condylar heads by a Morse’s cone attachment, either to facilitate intraoperative fitting to the glenoid prosthetic component or to allow future joint prosthetic replacement. The introduction of CAD/CAM technology for the design and manufacturing of patient-specific mandibular reconstruction plates, in combination with cutting and drill guide printing, has created a broad range of new opportunities for the planning and implementation of mandibular reconstruction. This concept can be applied not only to the reconstruction with bone grafts or bone flaps but also to stand-alone alloplastic reconstructions [22]. The need for condylar reconstruction while performing a mandibular replacement is a high debated topic in literature [31], due to postoperative pain and dysfunctional outcomes. One of the most used techniques reported in this literature review, is the articular disk sparing to prevent glenoid fossa resorption or bone surface anchylosis, sassociated with the shaping and the resuspension of the bone flap extremity, used as a new condyle (Table 2). In other case reports the articular prosthesis has been designed to be the replica of the condylar head. In the case we are reporting, the left condyle has been removed 5 years before during a previous surgical debulking, the right condyle was deformed, and the articular space was so thin revealing that the disk was malacic and could not be preserved. We decided that both condyles needed to be replaced by a bilateral TMJ patient-specific prosthesis, to obtain a good mandibular function and stability. We choose a round-shaped condyle. The patient has undergone radical surgery in 2021, and no major intra- or post-operative complications occurred, presenting good functional and aesthetic outcomes. On the 12^th^-month follow-up, the patient has undergone dental implant restoration revealing an optimal preservation and viability of the fibular bone flap, even if the free bone flap has no contact with other bones. To date, at the 18^th^-month follow-up, the occlusion has been restored by a dental implant-supported full arch prosthesis. The review of the literature on real total mandibular reconstructions demonstrates that the need for a total replacement of the mandible is fortunately a rare condition, mainly caused by osteomyelitis or by low-grade malignancies, but it's still a challenging topic in maxillofacial surgery for its functional and structural implications. The technological development in the field of maxillo-mandibular reconstruction, VSP and CAD-CAM techniques evolution, to date, justify a more extensive reconstructive approach, even towards benign diseases to achieve better cosmesis, improved function, and to reduce the disease recurrence rate.

## Conclusions

There are very few documented reports in the literature on total biarticular mandibular replacement. A new concept of hybrid bone and porous titanium mandibular implant for total mandibular replacement, has been presented achieving good short-term functional, aesthetic, and dental outcomes. Further clinical data must confirm its effectiveness, even if we have reached very interesting results, it is difficult to make definitive recommendations regarding the treatment of massive BFD of the mandible. More clinical data need to be added on this topic in future research works.

## Data Availability

Not applicable to this work.

## Abbreviations

CAD: Computer-aided design
CAM: Computer-aided manufacturing
VSP: Virtual surgical planning
FFF: Free fibular flap
RP: Rapid prototyping
BFD: Bone fibrous dysplasia
